# Reconstructing DNA methylation maps of ancient populations

**DOI:** 10.1093/nar/gkad1232

**Published:** 2024-01-23

**Authors:** Arielle Barouch, Yoav Mathov, Eran Meshorer, Benjamin Yakir, Liran Carmel

**Affiliations:** Department of Genetics, The Alexander Silberman Institute of Life Sciences, The Hebrew University of Jerusalem, Jerusalem 9190401, Israel; School of Computer Science and Engineering, The Hebrew University of Jerusalem, Jerusalem 9190401, Israel; Department of Genetics, The Alexander Silberman Institute of Life Sciences, The Hebrew University of Jerusalem, Jerusalem 9190401, Israel; Edmond and Lily Safra Center for Brain Sciences (ELSC), The Hebrew University of Jerusalem, Jerusalem 9190401, Israel; Department of Genetics, The Alexander Silberman Institute of Life Sciences, The Hebrew University of Jerusalem, Jerusalem 9190401, Israel; Edmond and Lily Safra Center for Brain Sciences (ELSC), The Hebrew University of Jerusalem, Jerusalem 9190401, Israel; Department of Statistics and Data Science, The Hebrew University of Jerusalem, Jerusalem 9190500, Israel; Department of Genetics, The Alexander Silberman Institute of Life Sciences, The Hebrew University of Jerusalem, Jerusalem 9190401, Israel

## Abstract

Studying premortem DNA methylation from ancient DNA (aDNA) provides a proxy for ancient gene activity patterns, and hence valuable information on evolutionary changes in gene regulation. Due to statistical limitations, current methods to reconstruct aDNA methylation maps are constrained to high-coverage shotgun samples, which comprise a small minority of available ancient samples. Most samples are sequenced using in-situ hybridization capture sequencing which targets a predefined set of genomic positions. Here, we develop methods to reconstruct aDNA methylation maps of samples that were not sequenced using high-coverage shotgun sequencing, by way of pooling together individuals to obtain a DNA methylation map that is characteristic of a population. We show that the resulting DNA methylation maps capture meaningful biological information and allow for the detection of differential methylation across populations. We offer guidelines on how to carry out comparative studies involving ancient populations, and how to control the rate of falsely discovered differentially methylated regions. The ability to reconstruct DNA methylation maps of past populations allows for the development of a whole new frontier in paleoepigenetic research, tracing DNA methylation changes throughout human history, using data from thousands of ancient samples.

## Graphical Abstract

**Graphical Abstract:**
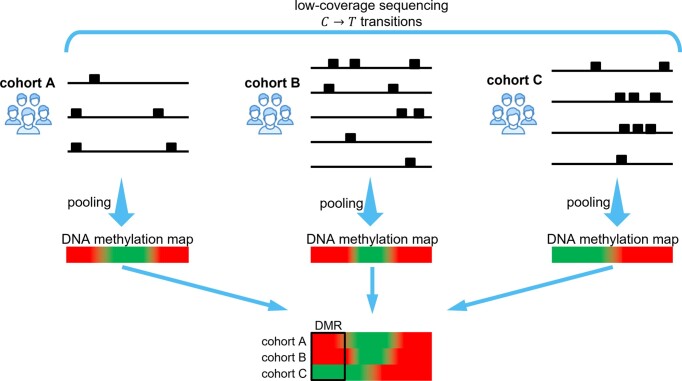

## Introduction

The ability to computationally reconstruct premortem DNA methylation maps in ancient humans (1–3) provided a useful tool to identify evolutionary changes in gene regulation. This opened up the field of paleoepigenetics (4,5) that was used to study regulatory changes that underlie phenotypic adaptations in humans. For example, comparing DNA methylation across human groups led to the identification of roughly 3000 differentially methylated regions (DMRs) separating Neanderthals, Denisovans, and modern humans (3). Analysis of these DMRs revealed that the vocal and facial anatomy is derived in modern humans and differs from that in Neanderthals and Denisovans (3). These DMRs were also used to predict anatomical features of the Denisovan, whose physical appearance has not yet been recovered in the fossil record (6).

The reconstruction method utilizes the fact that deamination, the predominant chemical decay process in ancient DNA (aDNA), works differently on methylated and unmethylated cytosines, turning the former into thymines and the latter into uracils (7). aDNA typically consists of double-stranded fragments with short single-stranded overhangs. The deamination rate varies substantially between these two regions. Single-stranded DNA can exhibit deamination rates as high as tens of percentages, while double-stranded DNA shows rates around 1% or lower (8). The overall average deamination rate across a fragment, when considering both regions, typically falls within the range of 1–3% (1,3). aDNA library preparation protocols that use full or partial USER (User Specific Excision Reagent) treatment trim the molecules at uracil positions but do not process thymines (7,9). This enables the fraction of thymines in positions where cytosines can be potentially methylated (CpG positions, where cytosines are followed by a guanine), known as the C$ \to$T ratio, to be used as a proxy for premortem DNA methylation level (1,2).

Paleoepigenetics holds great promise to shed new light on human evolution, but its applicability is limited because the reconstruction of aDNA methylation maps requires shotgun sequencing with a coverage of at least ∼15x. Such high-coverage samples, which also went through USER treatment, are relatively rare and comprise a Neanderthal (10), a Denisovan (11), and a few anatomically modern humans (AMHs) (3,12,13). In particular, reconstructing aDNA methylation is infeasible for the thousands of individuals sequenced using in-solution hybridization capture, which is the most widely used technology in the aDNA field. Hybridization capture probes target pre-determined loci, usually a large set of single-nucleotide polymorphic sites (SNPs). The most popular probe set used with aDNA is known as the 1240K set, targeting 1233013 SNPs and designed as a merger of the 390k set (targeting 394 577 SNPs) (14) and the 840k set (targeting 842 630 SNPs) (15). Hybridization capture enabled low-coverage sequencing of thousands of ancient individuals and was shown to provide much information on demographic events in past populations (16–18). Many key populations have been deeply investigated using 1240K hybridization capture, including the Neolithic and Bronze Age Europe (19), pre-contact Caribbeans (20,21), and Bronze Age Levant (22).

Here we show that although samples sequenced to low coverage using hybridization capture are unamenable to the reconstruction of DNA methylation, a combination of several samples from the same population can still provide a useful DNA methylation map. The key to our approach is the observation that DNA methylation patterns of individuals from the same population are more similar than DNA methylation patterns of individuals from different populations (23,24). This means that populations have characteristic DNA methylation profiles, in a similar way by which genetic profiles of populations can be defined based on genotypes. The differences in DNA methylation profiles between populations represent a combination of genotype differences, differences in shared environment and lifestyle, and random effects (25–28).

Instead of reconstructing individual DNA methylation maps, we develop here a method that pools samples together to obtain DNA methylation maps that represent entire populations. Combining the facts that each position may be sampled by multiple individuals, that each probe targets a region of approximately 100 base pairs around the target site (14,15), and that off-target sequences that capture non-target genomic regions are common, we show that the number of sampled ancient individuals in current studies is sufficient to provide a reliable reconstruction of genome-wide DNA methylation. We demonstrate the method on seven ancient populations and show that population-wise comparisons provide meaningful information and can be used to trace changes in DNA methylation throughout human history.

## Materials and methods

### Computation of $C \to T\;$ ratio

Reconstruction of DNA methylation proceeded according to the method described in Gokhman *et al.* (3), which is based on evaluating the $C \to T{\mathrm{\;}}$ratio in a position $i$ as ${t_i}/( {{t_i} + {c_i}} )$, where ${t_i}$ and ${c_i}$ are the counts of thymines and cytosines, respectively, in that position.

### Filtering out CpG positions

To remove possible PCR duplicates, coverage histogram was computed per chromosome per sample. After smoothing the histogram using a running window of length 5, we set the coverage threshold as the first coverage for which the histogram count was one or less, and then removed from the analysis all positions with coverage equal to or greater than the threshold.

CpG positions with a high rate of $C \to T{\mathrm{\;}}$ratio are likely to represent mutations rather than postmortem degradation. To filter them out, we removed positions whose $C \to T{\mathrm{\;}}$ratio exceeded 0.25 (1).

### Determining window size

We collect statistics on the ${t}$’s and ${n}$’s in a window of size $W$. Let $C$ be the effective coverage.

We require that the probability of observing a total of zero $t$’s in the window for a minimum methylation level ${m_0}$ is less than ${p_0}$. This translates into


\begin{eqnarray*}\Pr \left( {t = 0} \right) = {\left( {1 - \pi {m_0}} \right)^n} \,<\, {p_0}.\end{eqnarray*}

Taking log of both sides we get


\begin{eqnarray*}n \cdot \ln \left( {1 - \pi {m_0}} \right) \,<\, \ln {p_0},\end{eqnarray*}

meaning that we have to have a minimum accumulated coverage of


\begin{eqnarray*}n >\frac{{\ln {p_0}}}{{\ln \left( {1 - \pi {m_0}} \right)}}\end{eqnarray*}

in the window. If the window is covered by the average effective coverage, then $n = WC$. This translates into the following window size:


\begin{eqnarray*}W = \lceil \frac{1}{C} \cdot \frac{{\ln {p_0}}}{{\ln ( {1 - \pi {m_0}} )}}\rceil .\end{eqnarray*}

### Methylation in pooled samples

We tested two pooling procedures. For both, let us have $N$ samples, indexed by $i = 1, \ldots ,N$, and let us index the CpG positions by $j$.

Naïve pooling: Let ${c_{ij}}$ and ${t_{ij}}$ count the number of cytosines and thymines, respectively, at position $j$ in sample $i$. After pooling, the counts in this position are defined as the sum of counts in all samples,


\begin{eqnarray*}{c_j} = \mathop \sum \limits_{i = 1}^N {c_{ij}},{\mathrm{\;}}{t_i} = \mathop \sum \limits_{i = 1}^N {t_{ij}}.\end{eqnarray*}

Note that each sample has the same weight, regardless of potential difference in deamination rates or sequencing quality. After pooling, processing continues as if it were a regular single sample (3).

We used $C \to T$ ratio values to estimate methylation through the application of histogram matching (29). In essence, histogram matching is a technique that takes two datasets, called the *signal* and the *reference*, and devises a nonlinear transformation that—once applied to the signal—adjusts its histogram to be as close as possible to that of the reference. Let ${H_i}$, $i = 1, \ldots ,n$ be the cumulative histogram of the reference and ${h_j}$, $j = 1, \ldots ,m$ be the original cumulative histogram of the signal. Let ${B_i}$ be the values of the bins for histogram $H$, and ${b_j}$ be the values of the bins for histogram $h$. Then, we would like to find a transformation $t$ such that $b_j^{\prime} = t( {{b_j}} )$, and the cumulative histogram of the transformed signal, $h^{\prime}$, is as similar as possible to $H$. For each $j = 1, \ldots ,m$, we find the index ${i_0}$ where $H$ is most similar to $h$, namely


\begin{eqnarray*}{i_0} = \arg \min | {{H_i} - {h_j}} |.\end{eqnarray*}

Then, the transformation $t$ is defined as


\begin{eqnarray*}t( {{b_j}} ) = {B_{{i_0}}}.\end{eqnarray*}

To this end, we computed the nonlinear transformation of the $C \to T$ values that would produce a histogram that is as close as possible to a target histogram of measured DNA methylation in modern human bone (Bone2).

Advanced pooling: Let us consider a window around position $j$, which contains in individual $i$ a set ${P_{ij}}$ of informative CpG sites. We assume that $| {{P_{ij}}} | \ge 1$, otherwise this position is not further considered. We also assume the window is sufficiently small, such that the methylation level of its CpG sites in each individual $i$ are similar. Finally, we assume the population is homogeneous, and consequently denote the common methylation value in this window as ${m_j}$.

We assume that the observed number of thymine bases in window $j$ for sample $i{\mathrm{\;}}$is binomially distributed, ${t_{ij}}{\sim} B( {{n_{ij}},{m_j}{\pi _i}})$, where


\begin{eqnarray*}{n_{ij}} = \mathop \sum \limits_{p \in {P_{ij}}} {n_{ip}}\end{eqnarray*}

is the total number of reads covering the CpG positions in the window (${n_{ip}}$ is the number of reads that cover position $p$ in individual $i$), and ${\pi _i}$ is the deamination rate of individual $i$. The likelihood of individual $i$ is


\begin{eqnarray*}{L_{ij}} = \left( {\begin{array}{@{}*{1}{c}@{}} {{n_{ij}}}\\ {{t_{ij}}} \end{array}} \right){\left( {{m_j}{\pi _i}} \right)^{{t_{ij}}}}{\left( {1 - {m_j}{\pi _i}} \right)^{{n_{ij}} - {t_{ij}}}},\end{eqnarray*}

and the log-likelihood


\begin{eqnarray*}{\ell_{ij}} &=& {t_{ij}}\log \left( {{m_j}{\pi _i}} \right) \\ &&+ \left( {{n_{ij}} - {t_{ij}}} \right)\log \left( {1 - {m_j}{\pi _i}} \right) + {B_i},\end{eqnarray*}

where ${B_i}$ is a term that is independent of ${m_j}$. The total log-likelihood of all individuals is


\begin{eqnarray*} {\ell_j} &=& \mathop \sum \limits_{i = 1}^N {t_{ij}}{\mathrm{log}}\left( {{m_j}{\pi _i}} \right)\\ &&+ \mathop \sum \limits_{i = 1}^N \left( {{n_{ij}} - {t_{ij}}} \right){\mathrm{log}}\left( {1 - {m_j}{\pi _i}} \right) + B,\end{eqnarray*}

where $B = \mathop \sum \limits_{i = 1}^N {B_i}$ is a term independent of ${m_j}$. The score function with respect to ${m_j}$ is:


\begin{eqnarray*}\frac{{d{\ell_j}}}{{d{m_j}}} &=& \mathop \sum \limits_{i = 1}^N \frac{{{t_{ij}}}}{{{m_j}}} - \mathop \sum \limits_{i = 1}^N \frac{{\left( {{n_{ij}} - {t_{ij}}} \right){\pi _i}}}{{1 - {m_j}{\pi _i}}}\\ &=& \frac{{{T_j}}}{{{m_j}}} - \mathop \sum \limits_{i = 1}^N \frac{{\left( {{n_{ij}} - {t_{ij}}} \right){\pi _i}}}{{1 - {m_j}{\pi _i}}},\end{eqnarray*}

where


\begin{eqnarray*}{T_j} = \mathop \sum \limits_{i = 1}^N {t_{ij}}. \end{eqnarray*}

To obtain a maximum likelihood estimator of ${m_j}$ we equate the score function to zero and solve using Newton–Raphson. For this,


\begin{eqnarray*}\frac{{{d^2}{\ell_j}}}{{dm_j^2}} = - \frac{{{T_j}}}{{m_j^2}} - \mathop \sum \limits_{i = 1}^N \frac{{( {{n_{ij}} - {t_{ij}}} )\pi _i^2}}{{{{( {1 - {m_j}{\pi _i}} )}^2}}}. \end{eqnarray*}

Given $m_j^t$ is the approximate solution at iteration $t$, the solution at iteration $t + 1$ is given by


\begin{eqnarray*}m_j^{t + 1} = m_j^t - \frac{{d\ell/d{m_j}( {m_j^t} )}}{{{d^2}\ell/dm_j^2( {m_j^t} )}}. \end{eqnarray*}

In order to get an initial guess, we may obtain an approximated solution using


\begin{eqnarray*}\frac{1}{{1 - {m_j}{\pi _i}}} \approx 1 + {m_j}{\pi _i}. \end{eqnarray*}

Hence,


\begin{eqnarray*}\frac{{\ell_j}}{{d{m_j}}} \approx \frac{{{T_j}}}{{{m_j}}} - \mathop \sum \limits_{i = 1}^N ( {1 + {m_j}{\pi _i}} )( {{n_{ij}} - {t_{ij}}} ){\pi _i}. \end{eqnarray*}

This simplifies into


\begin{eqnarray*}\frac{{d\ell_j}}{{d{m_j}}} \approx \frac{{{T_j}}}{{{m_j}}} - \mathop \sum \limits_{i = 1}^N ( {{n_{ij}} - {t_{ij}}} ){\pi _i} - {m_j}\mathop \sum \limits_{i = 1}^N ( {{n_{ij}} - {t_{ij}}} )\pi _i^2. \end{eqnarray*}

Further approximating by neglecting terms of the order of $\pi _i^2$, we get


\begin{eqnarray*}\frac{{d\ell_j}}{{d{m_j}}} \approx \frac{{{T_j}}}{{{m_j}}} - \mathop \sum \limits_{i = 1}^N ( {{n_{ij}} - {t_{ij}}} ){\pi _i}. \end{eqnarray*}

This can be written as


\begin{eqnarray*}\frac{{d\ell_j}}{{d{m_j}}} \approx \frac{{{T_j}}}{{{m_j}}} - N_j^\pi + T_j^\pi ,\end{eqnarray*}

where


\begin{eqnarray*}N_j^\pi = \mathop \sum \limits_{i = 1}^N {\pi _i}{n_{ij}},{\mathrm{\;}}T_j^\pi = \mathop \sum \limits_{i = 1}^N {\pi _i}{t_{ij}}. \end{eqnarray*}

The approximate solution is therefore


\begin{eqnarray*}m_j^0 \approx \frac{{{T_j}}}{{N_j^\pi - T_j^\pi }}. \end{eqnarray*}

The Fisher information for estimating ${m_j}$ is equal to the expectation of the negative second derivative of the log-likelihood function,


\begin{eqnarray*}I\left( {{m_j}} \right) &=& - E\left( {\frac{{{d^2}\ell_j}}{{dm_j^2}}} \right) = E\left( {\frac{{{T_j}}}{{m_j^2}}} \right)\\ &&+ \mathop \sum \limits_{i = 1}^N E\left( {\frac{{\left( {{n_{ij}} - {t_{ij}}} \right)\pi _i^2}}{{{{\left( {1 - {m_j}{\pi _i}} \right)}^2}}}} \right).\end{eqnarray*}

The empirical Fisher information is the evaluation of this negative second derivative at the estimated value of the parameter, and may serve as an approximation of the Fisher information,


\begin{eqnarray*}\hat I\left( {{{\hat m}_j}} \right) = \left( {\frac{{{T_j}}}{{\hat m_j^2}}} \right) + \mathop \sum \limits_{i = 1}^N \frac{{\left( {{n_{ij}} - {t_{ij}}} \right)\pi _i^2}}{{{{\left( {1 - {{\hat m}_j}{\pi _i}} \right)}^2}}},\end{eqnarray*}

where ${\hat m_j}$ is the estimator obtained from the iterations of the Newton–Raphson algorithm. The empirical Fisher information is computed as part of the implementation of the algorithm. Finally, we may approximate the variance of the estimator via the inverse of the empirical Fisher information:


\begin{eqnarray*}V( {{{\hat m}_j}} ) \approx 1/\hat I( {{{\hat m}_j}} ).\end{eqnarray*}

### Calculating pooled sample deamination rate

A reference modern bone sample was used for this calculation, as done in Gokhman *et al.* (3). Only CpG positions in which methylation in the reference was one were included.

### Methylation in CpG islands and housekeeping genes promoters

We used the cpgIslandExt database for CpG islands (30,31), and a list of housekeeping genes (32), from which we created a list of promoter intervals (defined from 5000 upstream to 1000 downstream of the transcription start site).

### Predicting population effective coverage

We created a simple linear regression model that uses the number of pooled samples and their mean number of autosomal SNP hits as explaining variables, to predict the effective coverage of the pooled cohort. To create the model, we used data from the seven cohorts examined in this study. The resulting equation, obtained with $MSE = 0.85$ and ${R^2} = 0.99$, is


\begin{eqnarray*}E.{\mathrm{C}} &=& {\mathrm{\;}}0.2338 \cdot {\mathrm{NUM}}\_{\mathrm{SAMPLES}}\\ &&+ 5.04 \cdot {\mathrm{AVG}}\_{\mathrm{SNPS}} - 31.2914\end{eqnarray*}

A reasonable reconstruction can be obtained if the predicted effective coverage is above a threshold of 12 (Figure 2B).

### Computing correlations between DNA methylation maps

We computed correlations between DNA methylation maps as was suggested by Loyfer *et al.* (33) To this end, the genome was segmented to regions with roughly constant methylation and then correlations were computed based on the average methylation in these regions.

### DMRs filtration model

To assess the level of false DMRs between two populations, we generated 10 permutations over the population labels. DMRs detected for the permutated data are considered false discoveries. We scanned different values of ${\mathrm{\Delta }}$ and min_CpGs, and calculated the FDR per set of parameters by dividing the average number of DMRs found in the permutations by the observed number (Supplementary Table S12). We chose for further analysis parameter sets for which FDR < 0.05.

## Results

### Datasets

To test our approach to pool together different individuals to reconstruct DNA methylation maps of ancient populations, we used seven ancient cohorts, each consisting of at least 20 individuals sequenced using the 1240K hybridization capture. Each cohort represents a relatively homogeneous population, with the individuals coming from similar times and geographic locations. To avoid intra-population variability due to the tissue specificity of DNA methylation, the cohorts predominantly include individuals whose DNA was extracted from petrous bones. Individuals with <30 000 autosomal SNP hits were filtered out, as previous work showed that lower coverage might compromise inference reliability (22).

The seven cohorts are Baqah_BA (21 samples) from the Middle-to-Late Bronze Age in Baq’ah, Jordan (22); Cuba_ARC_CER (35 samples) from the Archaic and Ceramic Ages in Cuba (20,21); British_N (47 samples) from Neolithic England, Scotland and Wales (19); British_BA (51 samples) from Early, Middle and Late Bronze Age England, Scotland and Wales (19); Eu_BellBeakers (54 samples) from the Bell Beakers culture of central Europe (19); Mongolia_BA_IA (63 samples) from Bronze and Iron Ages Mongolia (34); and the Caribbean_CER population (142 samples) from the Ceramic Age in the Caribbeans (20) (Table 1, Supplementary Table S1).

**Table 1.** Basic information on the cohorts used in the current study

Population	Number of individuals	Period	Region	Average autosomal SNP hits	Average total CpG coverage	Effective Coverage; average total CpG coverage (excluding non covered CpGs)	Petrous to total ratio	Female to total ratio	References
Caribbean_CER	142	100–1500 CE	Caribbean	680 901	33.74	35.69	1.00	0.45	20
Mongolia_BA_IA	63	3000 BCE-100 CE	Mongolia	788 240	22.37	24.16	1.00	0.35	34
Eu_BellBeakers	54	2500–2000 BCE	Central Europe	671 619	12.02	14.29	0.98	0.30	19
British_N	47	4000–2500 BCE	UK	632 179	10.25	12.79	0.91	0.36	19
British_BA	51	2300–800 BCE	UK	605 833	9.47	12.00	0.96	0.49	19
Cuba_ARC_CER	35	1000 BCE - 1500 CE	Caribbean	656 432	6.80	9.44	1.00	0.43	20,21
Baqah_BA	21	1550–1150 BCE	Southern Levant	685 503	3.56	6.99	1.00	0.48	22

### Generating population-level ancient methylation maps

We generate a population methylation map by pooling together individuals from that population. To carry out pooling, we tested two approaches. The first, denoted here as the *naïve approach*, simply sums up the counts of cytosines and thymines in each CpG position from all samples. This approach is computationally fast but ignores potential differences in deamination rate across the pooled individuals. The second, denoted here as the *advanced approach*, is computationally more involved and does account for differences in deamination rates between individuals (see Methods). Comparing the two methods, we found that the *naïve approach* performs better, or at least equally as well (Supplementary Text T1). Therefore, hereinafter we will only present results using the *naïve approach*.

Following pooling, filtration proceeds similarly to the original reconstruction algorithm (1), with the main step being the removal of CpG positions with a C$ \to$T ratio above a threshold of 0.25, as they likely result from mutations and not deamination. Unlike the original reconstruction algorithm, the mapping from C$ \to$T ratios to methylation was performed by matching the histogram of the C$ \to$T ratios to the histogram of DNA methylation measured in modern human bone using whole-genome bisulfite sequencing (WGBS).

### Effective coverage of a population

**Figure 1.**
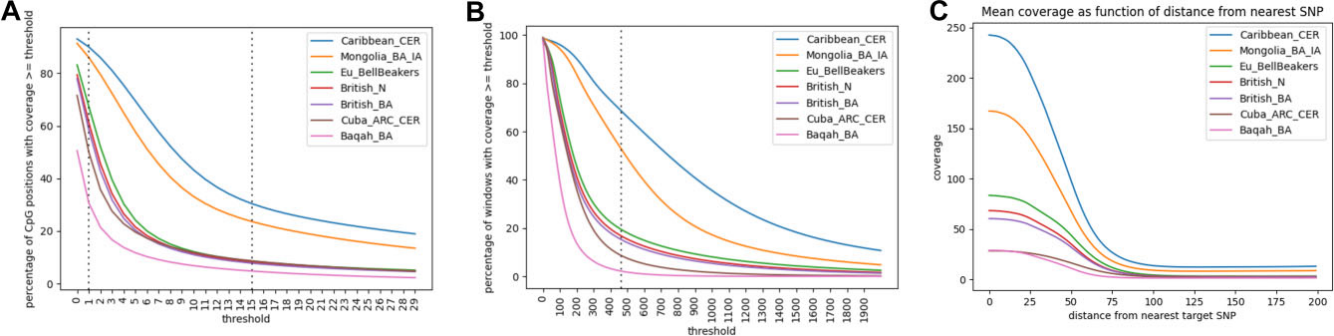
**(A)** Percentage of CpG sites with coverage larger than a certain threshold, for each of the pooled cohorts. **(B)** Percentage of windows (genomic regions spanning 31 consecutive CpG positions) with coverage larger than a certain threshold, for each of the pooled cohorts. **(C)** Average coverage as a function of the distance to the nearest target SNP.

We first wished to examine whether pooling produces C$ \to$T counts comparable to those obtained in high-coverage shotgun sequences. To this end, we looked at the fraction of CpG positions whose coverage is above a certain threshold (Figure 1A). Given the similar average number of autosomal SNP hits across the cohorts (Table 1), we expect that for a fixed threshold this fraction would increase with the number of samples in the cohort. Indeed, in Caribbean_CER, the largest cohort, 92.1% of the CpG positions are covered by at least one read, and 31.2% are covered by >15 reads. In contrast, in Baqah_BA, the smallest cohort, only 50.6% of the CpG positions are covered by at least one read, and 5.1% are covered by more than 15 reads.

For shotgun sequencing, DNA methylation in a particular CpG position is reconstructed using information from a window comprising neighboring CpG positions (1). Assuming a shotgun sample with low coverage of 15×, the use of window size of 31 CpG positions—the maximum value allowed in our reconstruction algorithm – achieves an average total coverage in the window of 465×. We therefore wanted to assess to what extent pooling achieves similar coverage levels, by computing the average coverage in windows of 31 consecutive CpG positions (Figure 1B). For the two largest cohorts, Caribbean_CER and Mongolia_BA_IA, 68% and 58% of CpG positions, respectively, are amenable to DNA methylation reconstruction using a window of size 31. This number drops to 2% for Baqah_BA (Supplementary Table S2). In comparison, the standard methylation arrays of 450K and EPIC (850K) provide information on 1.71% and 3.06% of CpG positions, respectively (35–37). Thus, in all cohorts, except Baqah_BA the number of CpG positions whose coverage allows for reliable reconstruction of aDNA methylation is larger than in these popular commercial arrays. A recent study claims that even shotgun coverage as low as 10x might suffice to reconstruct DNA methylation, using windows of up to 50 consecutive CpG positions (38). Using this estimate, we get that 75% of Caribbean_CER windows are amendable to reconstruction, and 1% of Baqah_BA (Supplementary Table S2). The two largest cohorts are the only ones for which the computed optimal window size is lower than 31. The optimal window sizes are 21 for Cermaic_CER and 29 for Mongolia_BA_IA, making 63% and 52% of the windows amenable to reconstruction, respectively (Supplementary Table S2).

To capture the coverage differences between cohorts, we define *the effective coverage* as the average coverage of all CpG positions in the genome that are covered at least once (Table 1). Effective coverage should increase with the number of samples in a population, and with the average autosomal SNP hits. In the current study, effective coverage changes monotonically with the number of samples, from 35.69 in Caribbean_CER to 6.99 in Baqah_BA. For comparison, we examined two high-coverage shotgun sequenced samples, Ust ‘Ishim (39) and the Altai Neanderthal (10), for which we computed effective coverages of 29.73 and 41.5, respectively.

As hybridization capture targets specific SNPs, we expect to see the coverage drops when we move away from the target site. To test this, we looked at the mean coverage as a function of the distance to the nearest target SNP in chromosome 1 (Figure 1C). Indeed, we observed a sharply decreasing curve, reaching a low plateau for distances above 100 bp, consistent with the fact that the hybridization probes target a sequence of roughly a hundred bases around each target site. The approximately constant coverage at distances higher than a hundred base pairs represents information coming from off-target sequences.

### Reconstructing DNA methylation of ancient populations

#### C→T ratio

**Figure 2.**
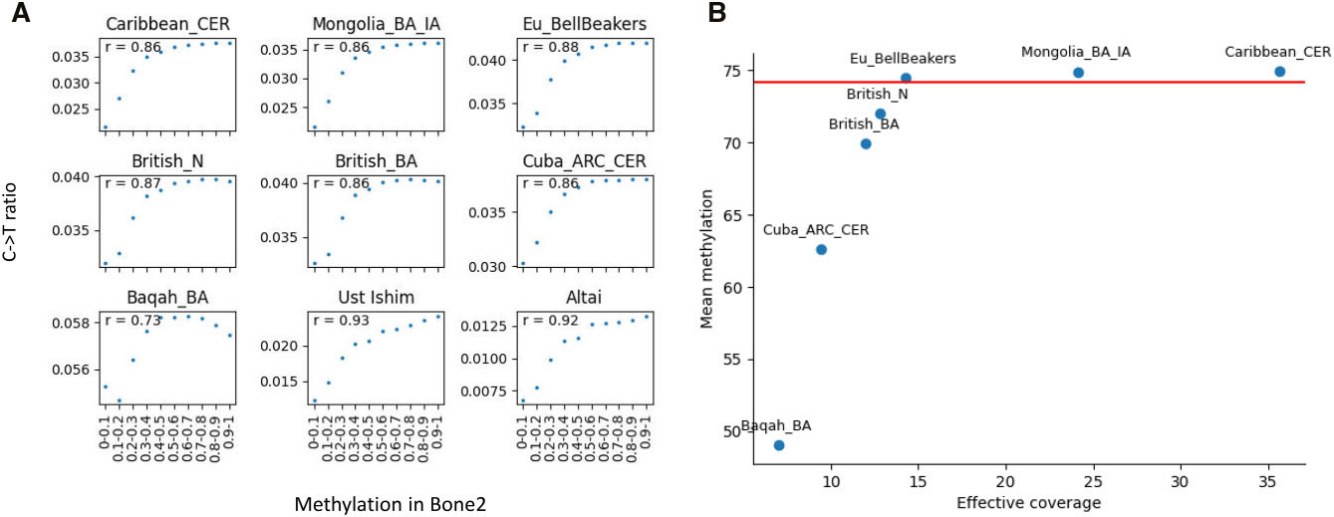
**(A)** C→T ratio in the pooled samples versus DNA methylation measured in modern human bone. CpGs were binned according to their measured methylation level in modern human bone. For each bin, an average C→T ratio was computed. Ust ‘Ishim (shotgun sequencing 42×) and the Altai Neanderthal (shotgun sequencing 52×) were added for comparison. ‘*r*’ denotes the Pearson correlation. **(B)** Mean methylation as a function of the effective coverage of the sample. Horizontal line shows mean methylation in Bone2.

The C$ \to$T ratio serves as a proxy for premortem DNA methylation, and has been used for the reconstruction of methylation in ancient samples sequenced using shotgun sequencing. We wanted to confirm that the C$ \to$T ratio computed on pooled samples may also serve as a proxy for aDNA methylation. To this end, we divided the CpG positions in the genome into ten bins, based on their methylation level in modern human bone (Bone2 (3)). Then, we computed the average C$ \to$T ratio across the positions in a bin, for each of the cohorts. As a reference, we repeated these computations for Ust ‘Ishim and the Altai Neanderthal. With the exception of the smallest cohort, Baqah_BA, the C$ \to$T ratio was monotonically increasing as a function of modern DNA methylation, attesting to its appropriateness to serve as the basis for reconstructing premortem DNA methylation (Figure 2A, Supplementary Text 2). The nonlinearity between the $C \to T$ ratio and the reference methylation is accounted for by a histogram matching procedure (see Methods). Moreover, again with the exception of Baqah_BA, the correlation between the C$ \to$T ratio and the binned methylation was in the range 0.86–0.88, similar to the correlation of 0.92 for the Altai Neanderthal and 0.93 for Ust ‘Ishim. Note that the range of C$ \to$T values varies across samples, due to variations in deamination rates and effective coverages. The outlying characteristics of Baqah_BA are a result of the small size of this cohort, suggesting that a cohort with an effective coverage as low as 6.99 provides insufficient information on premortem DNA methylation. In the next section, we will determine a more accurate bound on the minimum effective coverage required for a reliable reconstruction of DNA methylation in pooled cohorts.

#### The reconstruction algorithm

As we did for shotgun sequenced samples, we take advantage of the high correlation in methylation levels between neighboring CpGs (40) to reduce the standard error of our estimated methylation values. To this end, we define a window of a fixed number of consecutive CpG positions and estimate the DNA methylation in a CpG position using information from all CpG positions in the window centered around it. We set the size of the window adaptively, based on the effective coverage of the cohort, but bounding it from above by 31 CpG positions (see Materials and methods). As mentioned above, all cohorts reached this upper bound, except for the two with the highest effective coverage – Caribbean_CER with a window size of 21, and Mongolia_BA_IA with a window size of 29. The number of informative CpGs within a window tends to be smaller in the low-quality samples (Supplementary Figure S1). Premortem DNA methylation was computed from the C$ \to$T ratio using *histogram-matching* (29) to obtain a reconstructed DNA methylation histogram as similar as possible to that of a reference histogram in modern bone (Supplementary Figure S2).

About 75% of CpG positions are methylated throughout the mammalian genome (41). Specifically, the average methylation in our reference modern human bone is 74.15%. To test how the different cohorts replicate this characteristic value, we measured the average methylation per cohort (Figure 2B). All populations showed average methylation between 70% and 75%, with the exception of Baqah_BA and Cuba_ARC_CER, which have the smallest number of samples and the lowest effective coverage. More generally, the average DNA methylation of the population is a monotonic increasing function of the effective coverage, which allowed us to set an effective coverage of 12 as a minimum threshold for reconstructing population DNA methylation (Figure 2B).

To obtain a quick way to evaluate whether the effective coverage of a cohort is sufficient to allow for the reconstruction of aDNA methylation, we created a linear model that predicts the effective coverage of a population based on the number of individuals in the cohort and the average number of autosomal SNP hits (Supplementary Table S3), two simple and readily accessible variables. The linear model has $MSE = 0.85$ and ${R^2} = 0.99$ (see Materials and methods).

**Figure 3.**
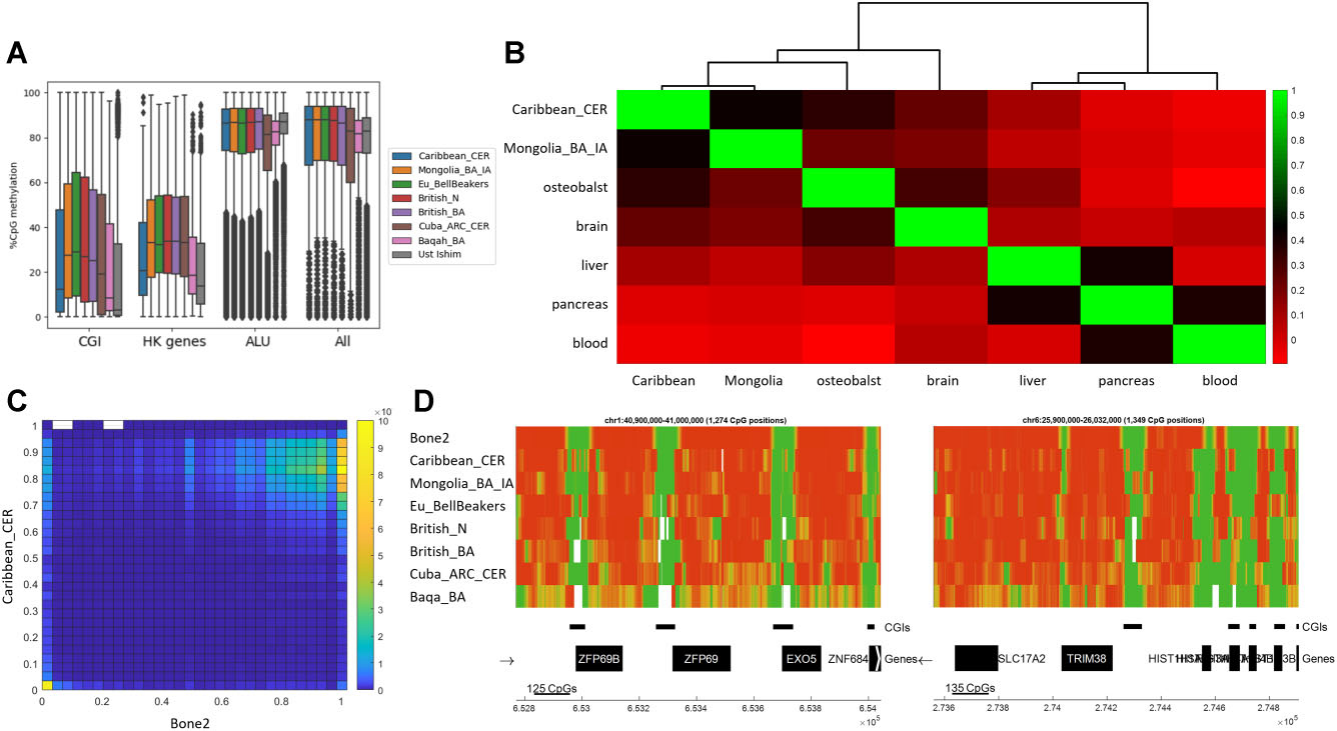
**(A)** DNA methylation in CGIs, housekeeping genes promoters, ALU elements and across the genome. **(B)** Pearson correlations between the reconstructed DNA methylation of Caribbean_CER and Mongolia_BA_IA, and modern DNA methylation in human tissues and cell types. The ancient populations cluster with osteoblast. Correlations were computed based on genome segmentation, see Materials and methods. **(C)** Heatmap of Bone2 methylation versus Caribbean_CER methylation with 50 bins per axis. Match between these two methylation maps is manifested by the hot regions around high and low methylation levels. **(D)** Ribbon plots showing measured and reconstructed DNA methylation patterns in two random genomic regions. Methylation is color coded from low methylation in green to high methylation in red.

To further validate the accuracy of our reconstructed DNA methylation, we conducted four analyses to assess the similarity between our reconstructed DNA methylation maps and the modern human bone map. First, we measured the mean methylation in the promoters of housekeeping genes and within CpG islands (CGIs), two genomic regions that are known to be strongly hypomethylated (42). We computed the average DNA methylation in these regions in our cohorts, as well as in a modern bone sample and the high-coverage shotgun sample Ust'-Ishim (Figure 3A). As expected, the DNA methylation within CpG islands and housekeeping gene promoters is significantly lower than the genomic average in all populations (Supplementary Table S4), with Caribbean_CER showing trends that most resemble modern DNA methylation. Similarly, we measured the mean methylation within Alu elements, which are transposable elements known for their high density of CpG positions and high methylation (43). As expected, the average DNA methylation levels withing these elements are high, consistent with the levels observed across the entire genome (Figure 3A).

Next, we computed correlations between the reconstructed pooled DNA methylation maps of the cohorts and the measured maps in several human tissues and cell types, including osteoblasts (33). As expected, given the skeletal origin of aDNA, our populations showed the greatest resemblance to osteoblast (Figure 3B). We then further demonstrated the match between the reconstructed pooled methylation and bone methylation by generating heatmaps that compare the histograms of Bone2 and the reconstructed cohort methylation (Figure 3C). Finally, we produced ribbon plots for random genomic segments, offering a visual testament of the high quality of the reconstructed methylation, particularly for cohorts with sufficiently high effective coverage (Figure 3D).

### DMRs detection

#### Methylation in known DMRs

**Figure 4.**
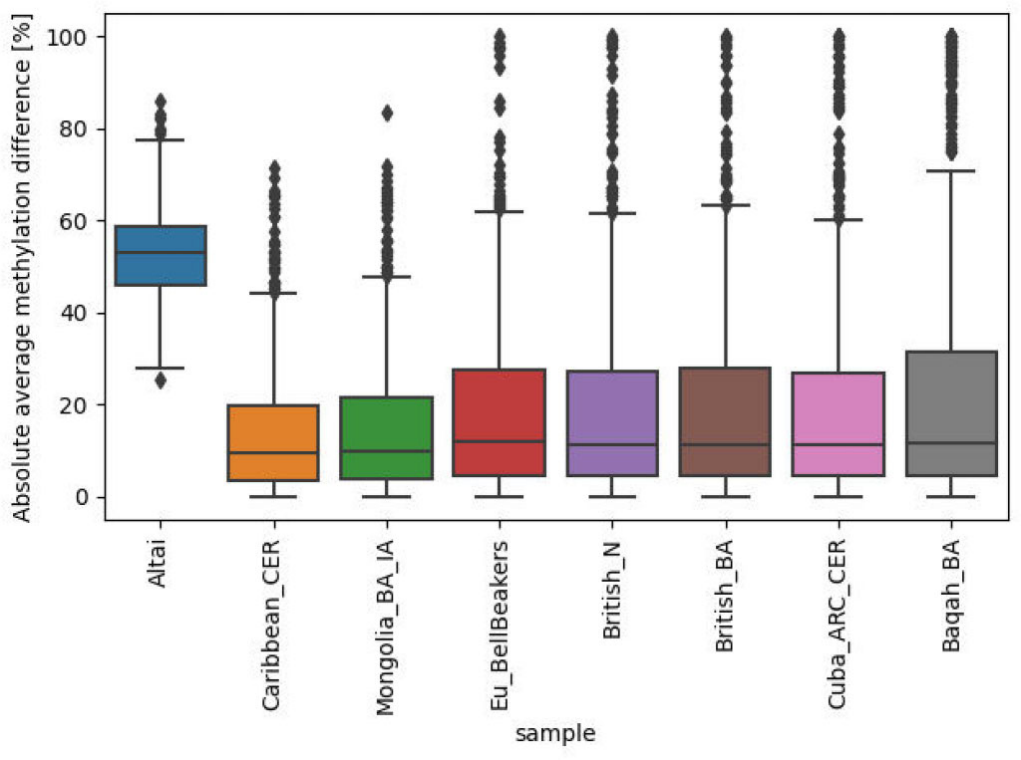
Absolute value of the difference between the average methylation in the cohort and the average methylation in Ust ‘Ishim, for the 873 modern human-derived DMRs.

In a previous study based on reconstructed DNA methylation in high-coverage shotgun samples, we identified 873 differentially methylated regions (DMRs) that are derived in modern humans, meaning that the methylation change occurred in the lineage leading to modern humans after they split from Neanderthals and Denisovans (archaic humans) (3). To further validate the quality of the reconstructed DNA methylation in the seven cohorts, we tested whether the average methylation in these DMRs in each of the cohorts is consistent with the methylation patterns observed in modern humans. For each DMR, we measured the distance between its average methylation in each of the cohorts and its average methylation in Ust'-Ishim. For comparison, we measured these distances also for the Altai Neanderthal. Reassuringly, the median of the distances was around 7% for all cohorts, whereas it was around 53% for the Altai Neanderthal (Figure 4).

#### De-novo detection of DMRs

We next wished to use the cohort DNA methylation profiles to detect DMRs separating modern and archaic humans and compare them to DMRs detected using high-coverage shotgun samples (3). For this, we first ran the DMR detection algorithm for each of the cohorts, excluding Cuba_ARC_CER and Baqah_BA, which have the lowest effective coverage, against a group of archaic humans consisting of the Altai Neanderthal (10) and a Denisovan (11). We then reran the DMR detection algorithm comparing a group of high-coverage shotgun samples of anatomically modern humans to the same group of archaic humans. The group of anatomically modern humans included SF12 (12) (Sweden, 9033–8757 before present (BP), femur, 73.0×), I1 583 (3) (Turkey, ∼8500 BP, petrous, 32.3×), Ballito Bay A (13) (South Africa, 2000–1960 BP, petrous, 14.3×) and Eland Cave (13) (South Africa, 510–450 BP, tibia, 11.8×). Based on the high quality of the shotgun samples, we treated the DMRs detected using them as a reference set of DMRs, to which we compared the DMRs detected using the pooled cohorts.

In all comparisons, DMR detection was carried out using the same set of parameters, including a methylation difference threshold (${\mathrm{\Delta }}$) of 0.4 and a minimum number of CpG positions per DMR of 50 (3). We divide the DMRs into three categories: *Shared-DMRs* are DMRs detected in both analyses; *cohort-DMRs* are DMRs detected only using the pooled cohorts and are thus potentially false positives; and *shotgun-DMRs* are DMRs detected only using the shotgun samples and are thus potentially false negatives. Using these approximations, we computed for each cohort the recall and precision, as well as the *F*1-score that combines both measures (Supplementary Table S5). The performance of the DMR detection algorithm highly depends on the quality of the samples, with Caribbean_CER and Mongolia_BA_IA having the highest *F*1-score. The lower-quality cohorts exhibit lower recall and precision due to a large number of false positives and false negatives, likely due to higher levels of noise in the methylation reconstruction process. Repeating the DMR detection using all five cohorts pooled together as representing modern humans, we obtained a mild improvement in recall, precision and *F*1-score (Supplementary Table S5).

To reduce the number of false positives, we sought to further filter the DMRs detected using the pooled cohorts. To this end, we characterized each DMR by six features: its total length in bases, its total number of CpG positions, the average methylation difference between the compared groups (${\mathrm{\Delta }}$), its maximum ${Q_t}$, its mean coverage and its genomic context (whether it is within a CpG island, gene promoter, or gene body). For all cohorts and for each feature but ${\mathrm{\Delta }}$, the shared-DMRs differed significantly from the cohort-DMRs, suggesting that these features might be useful in discriminating shared-DMRs from cohort-DMRs (Supplementary Table S6). Indeed, we were able to develop a classifier that filters out DMRs that are likely cohort-DMRs, and by improving precision on the expense of recall, we were able to considerably reduce false detection of DMRs (Supplementary Text T3, Supplementary Tables S7 and S8).

### Detecting DMRs between populations

Finally, we wanted to apply the DMR detection pipeline developed above to identify DMRs separating the different cohorts. We ran the DMR detection algorithm between pairs of populations, using ${\mathrm{\Delta }} = 0.4$ and a minimum of 40 CpG positions within a DMR. These parameters are less strict than in the previous section, to allow the detection of subtler DNA methylation changes (Supplementary Tables S9 and S10; Supplementary Text T4).

When comparing two populations, we reasoned that a better way to filter falsely detected DMRs is to carry out permutations of the population labels, and choosing parameter values that guarantee a false discovery rate less than a certain threshold (see Materials and methods, Supplementary Table S11). Comparing Mongolia_BA_IA to European populations, we detected 13 DMRs, which are present in the promoter or gene body of 13 genes. One of the genes is *MEIS1*, which is a major regulator of limb development, and is associated with myeloid leukemia. It was shown to be downregulated by promoter methylation in leukemia (44), and was also found to have gone through methylation changes separating modern from archaic humans (1). Another example is *RUNX2*, which plays a role in osteoblast differentiation (45). Comparing Mongolia_BA_IA to Caribbean_CER, we detected two DMRs, and another two were detected when comparing Caribbean_CER to European populations. Finally, comparing European populations to populations of Asian origin (Mongolia_BA_IA and Caribbean_CER), we found eight DMRs, including one inside the promoter region of *IFITM3*, which encodes an antiviral protein, and which shows lower methylation in its promoter in EV71-HFMD (Hand, foot and mouth disease induced by enterovirus 71) patients (46). Notably, polymorphism in the SNP rs12252 within the gene was found to be associated with susceptibility to COVID-19, and its mutant allele was found to be highly frequent in East Asian patients and rare in European patients (47).

Variation in bone type composition and sex ratio between cohorts have the potential to introduce confounding factors affecting the detection of DMRs between populations. Bone type composition is not a source of concern in our data, as the vast majority of individuals were sampled from petrous bones (Table 1). However, sex ratio differs between cohorts, with three showing a bias toward males (Table 1).

## Discussion

Our study has demonstrated that it is possible to reconstruct aDNA methylation patterns even when high-coverage samples are not available. Our work is based on the premise that variability in DNA methylation is lower within, compared to between, populations. We therefore reasoned that we might reconstruct DNA methylation profile characteristic of a past population by pooling together the DNA sequences of a sufficient number of low-coverage ancient individuals from that population. Indeed, we were able to generate high-quality methylation maps of past populations that were later used to detect differential methylation. As the vast majority of ancient samples are sequenced to low-coverage, our study expands the scope of aDNA methylation studies, allowing them to consider populations that have so far not been used.

The ability to reconstruct DNA methylation of past populations, and the constantly increasing density of ancient samples from diverse locations and times, would allow for spatiotemporal mapping of changes in DNA methylation. Combined with the high responsiveness of DNA methylation to environmental cues, such a spatiotemporal mapping would enable the use of aDNA methylation to study past environments (25).

Here, we analyzed seven populations, whose members were sequenced using the popular 1240K in-situ hybridization capture. However, the methodology we have developed is completely general and should work also on samples sequenced with different types of hybridization capture sets, as well as on low-coverage shotgun samples.

As expected, a DNA methylation map reconstructed using many low-quality samples inherently contains more noise compared to a map reconstructed from a high-coverage sample. We concluded that to achieve a reliable reconstruction, the effective coverage of the cohort should be at least 12, but preferably exceeding 15. We presented a straightforward regression model that enables the prediction of the effective coverage of a cohort prior to downloading the data, based solely on the number of samples and the mean number of SNP hits. This model has the potential for refinement with the future inclusion of additional populations.

The increase in noise is manifested by an increased rate of falsely detected DMRs. In the current study, we used two setups to test the quality of DMR detection using population DNA methylation maps, and considered means to reduce the number of falsely detected DMRs in both. First, we tried to compare a population with high-coverage individuals. In such a setup label permutation is infeasible, and we have therefore devised a machine-learning algorithm that filters out DMRs that are likely false positives. Whereas this allowed for a considerable improvement in the rate of falsely detected DMRs, this rate may still be high in some applications, and we generally do not recommend using such a setup unless the population is characterized by very high effective coverage. Second, we compared populations to each other, in an attempt to identify DMRs separating the different populations. Here, label permutation can be used, and DMR detection parameters can be adjusted to achieve a low false discovery rate. We highly recommend using population DNA methylation maps in such a setup, and the resulting small number of DMRs testify for the efficiency by which the number of falsely detected DMRs is reduced. For example, contrasting European and Asian populations revealed a DMR within the promoter of *IFITM3*, potentially related to differential viral exposure of these populations, and reflecting different frequencies of *IFITM3* allele in East Asians compared to Europeans.

Whereas the majority of population studies are based on petrous bones, high-coverage samples came from a variety of skeletal parts, including tibia and femur. This provides further advantage to detecting DMRs separating different populations, rather than DMRs separating populations from high-coverage samples, the latter being potentially affected by biases introduced by the use of different skeletal parts.

It is interesting to note that despite of the fact that naïve pooling does not take into account potential differences in deamination rate across individuals, it still performs better than the advance pooling technique (Supplementary Text T1). This suggests that differences in deamination rate are small compared to other sources of variance between the samples, especially in light of the fact that individuals from approximately the same place and time should have gone through similar deamination processes.

It is worth highlighting that in-situ hybridization capture sequencing, while designed to target specific genomic positions, exhibits notable presence of off-target reads. In fact, after filtering out reads with low mapping quality, approximately 65% of the covered CpG positions originate from off-target reads (Supplementary Figure S3).

## Supplementary Material

gkad1232_Supplemental_Files

## Data Availability

No new data were generated or analyzed in support of this research.
