# Supplementary material for: Reconstructing DNA methylation maps of ancient populations
Source: Nucleic Acids Res. 2024 Jan 23;52(4):1602–12. doi: 10.1093/nar/gkad1232 (PMC10939417; doi:10.1093/nar/gkad1232)
Supplement: gkad1232_Supplemental_Files [file gkad1232_supplemental_files.zip › Supplementary Figures.pdf]

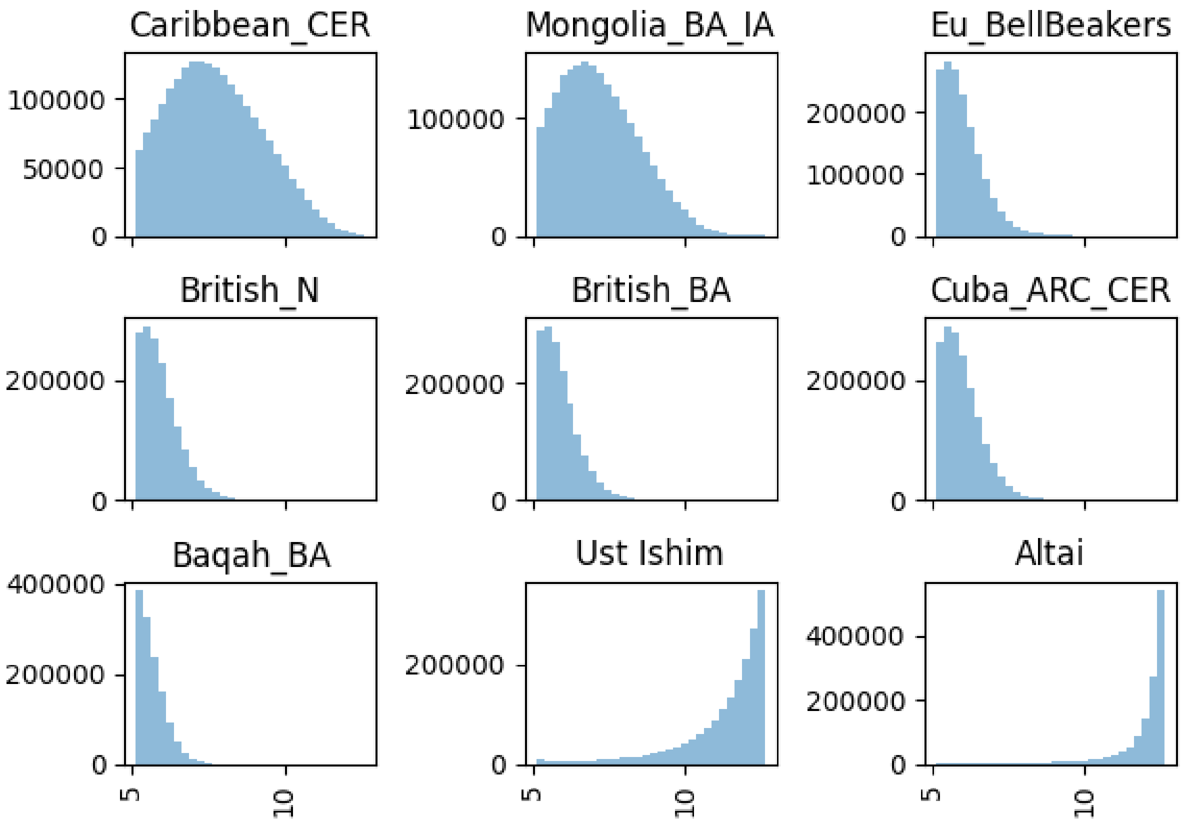

Supplementary Figure S1. The number of informative CpGs (covered by at least 15 reads) within a window of size 31. Data presented for all cohorts, as well as for the shotgun samples Ust 'Ishim and the Altai Neanderthal.

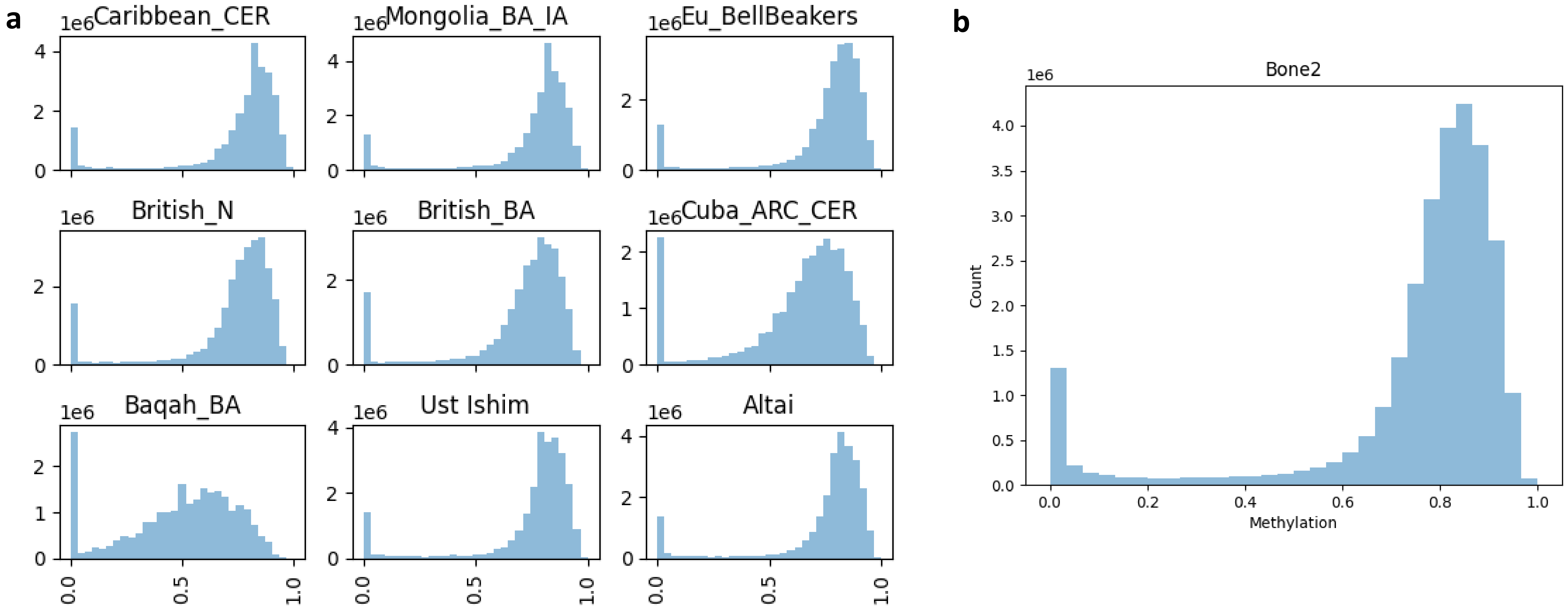

Supplementary Figure S2. DNA methylation was computed using histogram matching to the Bone2 reference. **a)** Histograms of reconstructed DNA methylation in the pooled cohorts, as well as in Ust 'Ishim and the Altai Neanderthal. **b)** Histogram of measured DNA methylation in Bone2, used as the reference in the histogram-matching.

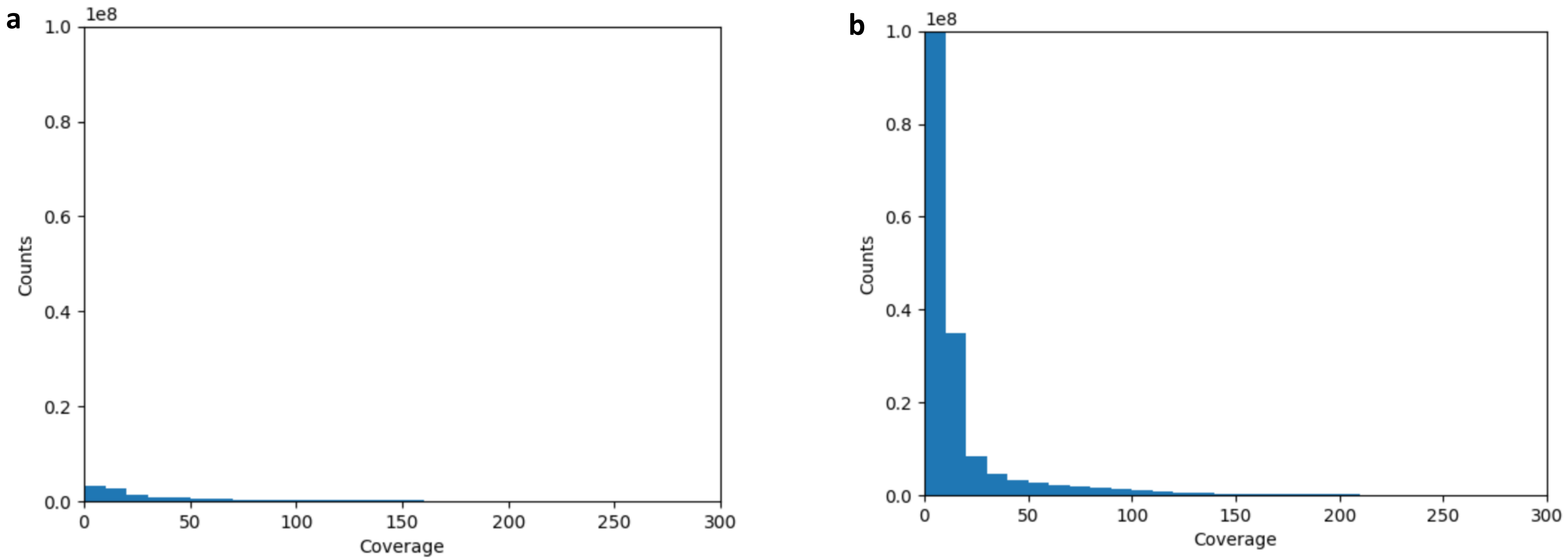

Supplementary Figure S3. Histogram of coverage of CpG positions in the Caribbean_CER cohort of **a)** for on-target, and **b)** off-target, reads.
