# Supplementary material for: Reconstructing DNA methylation maps of ancient populations
Source: Nucleic Acids Res. 2024 Jan 23;52(4):1602–12. doi: 10.1093/nar/gkad1232 (PMC10939417; doi:10.1093/nar/gkad1232)
Supplement: gkad1232_Supplemental_Files [file gkad1232_supplemental_files.zip › Supplementary texts.pdf]

Supplementary Texts for the paper:

Reconstructing DNA methylation maps of ancient populations

By Arielle Barouch et al.

Supplementary Text T1

Two approaches were tested for pooling samples for the purpose of reconstructing DNA methylation representative of a population, denoted as the naïve approach and the advanced approach (see Methods). Here, we compare the performance of the two methods and explain why we selected the naïve approach as the basis for analyses in the current study.

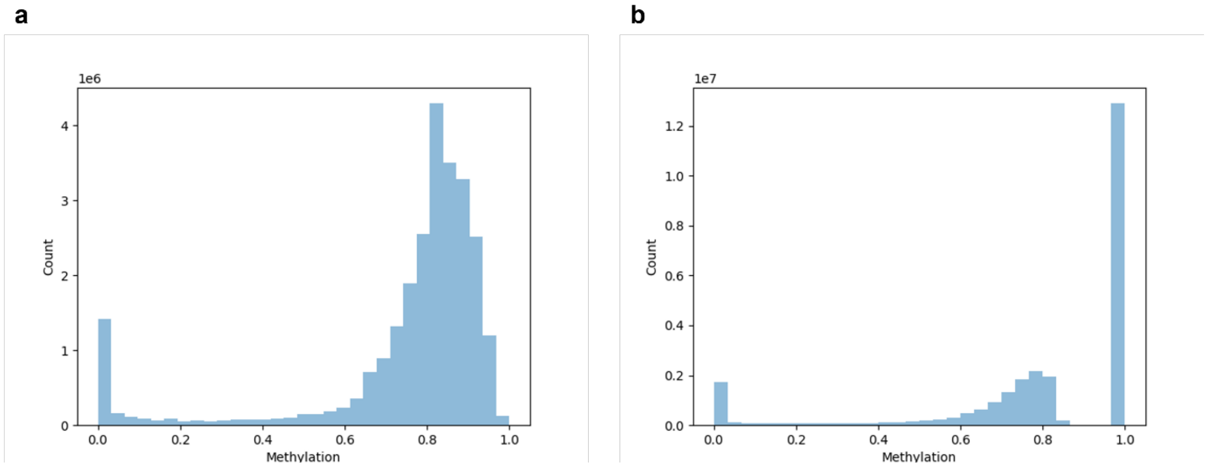

Figure T1.1. *Histogram of DNA methylation produced for Caribbean_CER for* ***a****) the naïve approach, and* ***b****) the advanced approach.*

For the comparison we reconstructed the methylation of the largest of our cohorts, the Caribbean_CER population, using both approaches, and compared various characteristics of the resulting DNA methylation maps. The overall mean methylation was 74.9% using the naïve approach, 79.6% using the advanced approach, and 74.2% in the modern reference Bone2. Therefore, the naïve approach provides a closer estimation of the expected value.

We also compared the histograms of DNA methylation across the two methods. While both produce the expected bimodal histograms that peak around low and high methylation levels, the advanced approach produced a much patchier histogram (Figure T1.1).

Lastly, we compared the distribution of methylation levels in CpG islands and housekeeping gene promoters (Figure T1.2). Again, the distribution of methylation in both CpG islands and housekeeping gene promoters showed DNA methylation levels closer to Bone2, compared to the advanced approach.

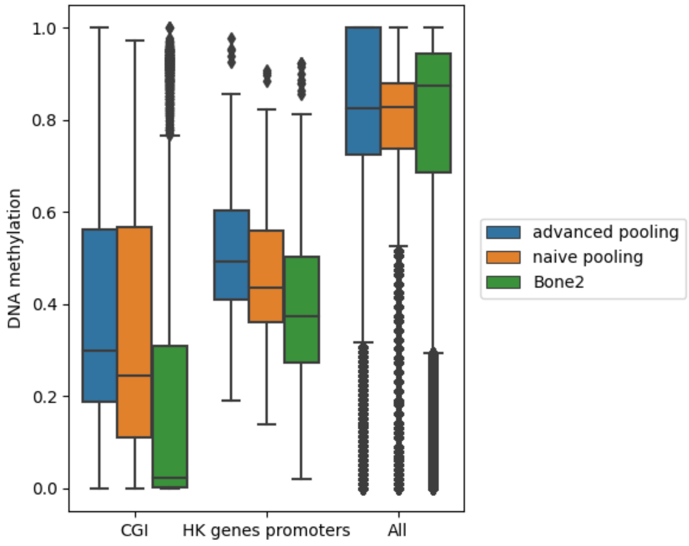

Figure T1.2*. Box plots of DNA methylation values in CGIs, housekeeping genes promoters and across the genome comparing the naïve and advanced methylation reconstruction methods in Caribbean_CER.*

Supplementary Text T2

In Gokhman et al. (Science 2014) we employed a reduced representation bisulfite sequencing (RRBS) sample as the reference for DNA methylation in modern bone, as it was the only available sample at the time. RRBS is known for providing information on ~12% of CpG positions, but is heavily biased towards CpGs residing within CpG islands and other GC-rich regions. Since this work, our lab has produced the first whole-genome bisulfite sequencing (WGBS) of modern human bone, offering a genome-wide unbiased assessment of DNA methylation in modern human bone[1].

To produce the plots shown in Figure 2a, we used these WGBS data as the reference for binning. As a result of this change in reference methylation, these plots show reduced correlations between average $C \rightarrow T$ values and modern beta values compared to Gokhman *et al*. (Science 2014). To show this, we have included here similar plots to those in the manuscript, using RRBS instead of WGBS as the reference. Indeed, the plots show better linearity and increased levels of correlations, akin to those observed in Gokhman *et al*. (Science 2014).

In addition, in Gokhman *et al*. (Science 2014), we used a maximum-likelihood approach to infer methylation from $C \rightarrow T$ values, which resulted in a truncated linear transformation. Here, to account for the non-linearity seen in Figure 2a, we have replaced the maximum-likelihood estimator with a histogram matching procedure (see Methods).

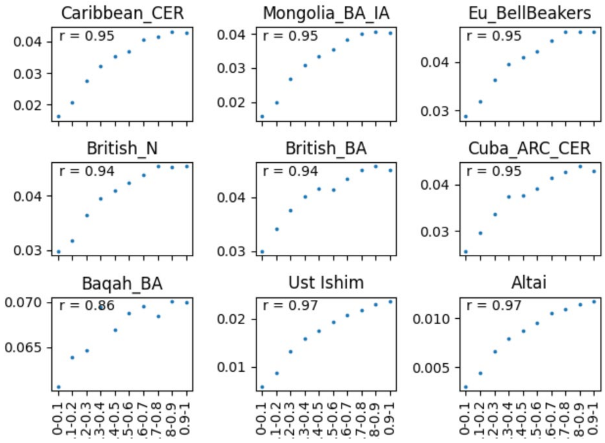

***Figure T2**.1. C→T ratio in the pooled samples versus DNA methylation measured in modern human bone. CpGs were binned according to their measured methylation level in an RRBS sample of modern human bone. For each bin, an average C→T ratio was computed. Ust 'Ishim (shotgun sequencing 42x) and the Altai Neanderthal (shotgun sequencing 52x) were added for comparison. "r" denotes Pearson correlation.*

Supplementary Text T3

To discriminate shared-DMRs from cohort-DMRs, we built a classification model based on the six features that characterize DMRs: total length in bases, total number of CpG positions, average methylation difference between the compared groups (Δ), maximum Q_t, mean coverage and genomic context (see main text). We tried several classification algorithms, including polynomial SVM, logistic regression, random forest and naïve Bayes, using the python package *sklearn* (Figure T2.1). The penalty used for the logistic regression was L2. The random forest classifier hyperparameters were 50 trees, 3 splits, 3 max features and 2 max depth. The SVM classifier has a polynomial kernel, where its kernel coefficient (γ) was calculated by the number of features, and the penalty parameter C was set to 1. To tune the hyperparameters for the SVM and Random Forest models, we used *sklearn*'s GridSearchCV, which performs exhaustive run over combinations of hyperparameters, attempting to optimize a score function, in our case the average precision defined as:

$$AP = \sum_n (R_n - R_{n-1}) P_n.$$

Here, P_n and R_n are the precision and recall, respectively, for the nth acceptance threshold. The acceptance threshold defines how to map the output of the model, a probability, to a binary category. The AP also accounts for the area under the recall-precision plot.

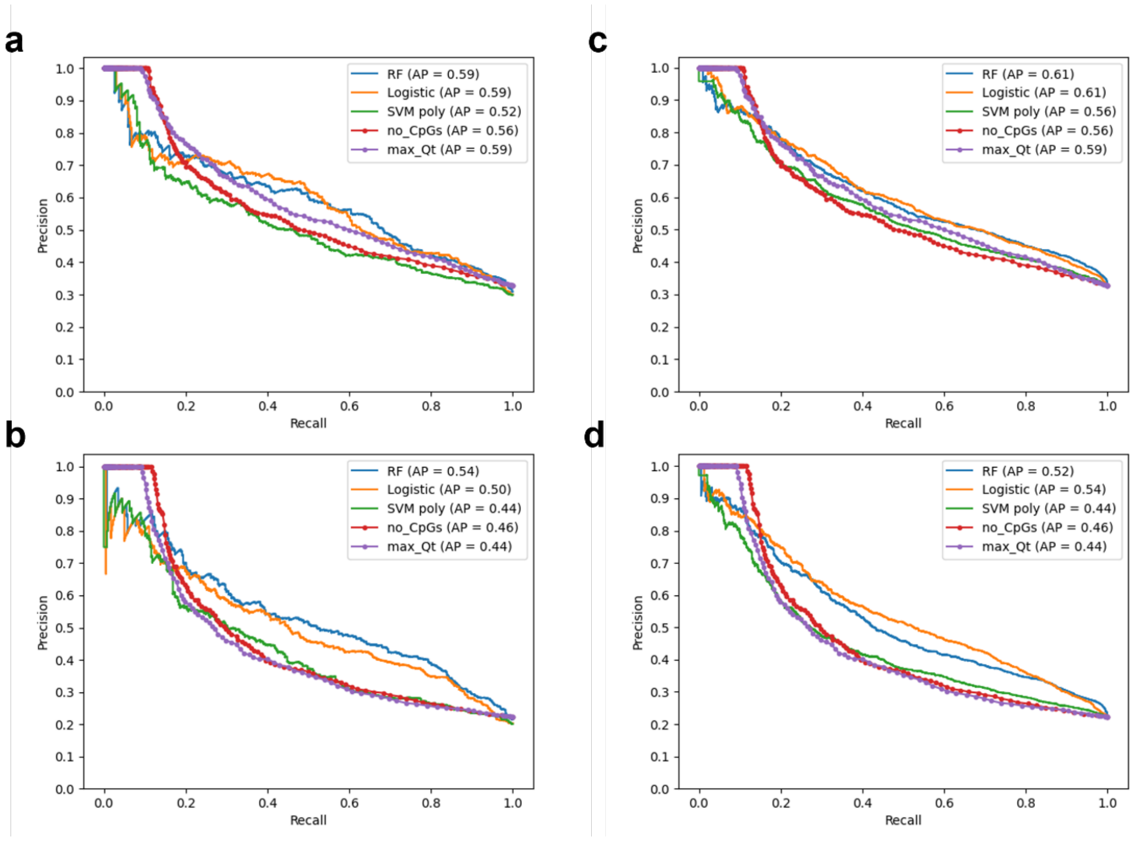

Figure T3.1 *Recall-precision plots for different classification algorithms. Using chromosomes 4-22 as a train set and chromosomes 1-3 as a test set for* ***a****) Mongolia_BA_IA, and* ***b****) Eu_BellBeakers. Using Caribbean_CER labeled DMRs as train set for* ***c****) Mongolia_BA_IA, and* ***d****) Eu_BellBeakers*

In addition to these classification algorithms, we tested a simple classification based on applying a threshold to a single parameter. For this, we used the two most discriminatory features, namely the maximum Q_t, and the number of CpG positions.

To create train and test sets, we used two different approaches. The first used the shared-DMRs and cohort-DMRs in chromosomes 5-22 as the train set, and those in chromosomes 1-4 as the test set (Supplementary Table S7, Methods). The second used different cohorts as train and test sets, including downsampled populations (Supplementary Table S8, Methods). There was no significant difference in performance between both approaches. In both, the best-performing algorithms were logistic regression and random forest, and the most significant explaining variable was the effective coverage. We noticed that in the second approach, a model performance was insensitive to the choice of the train set (Supplementary Table S8). Choosing different acceptance thresholds changes the balance between precision and recall (Figure T2.2).

Simple classification that used a threshold on the maximum Q_t performed surprisingly well for the high-quality cohorts, with AP = 0.61 for Caribbean_CER (the best performing model for this population, logistic regression, had AP = 0.64), and AP = 0.59 for Mongolia_BA_IA (the best performing model for this population, logistic regression, scored AP = 0.62).

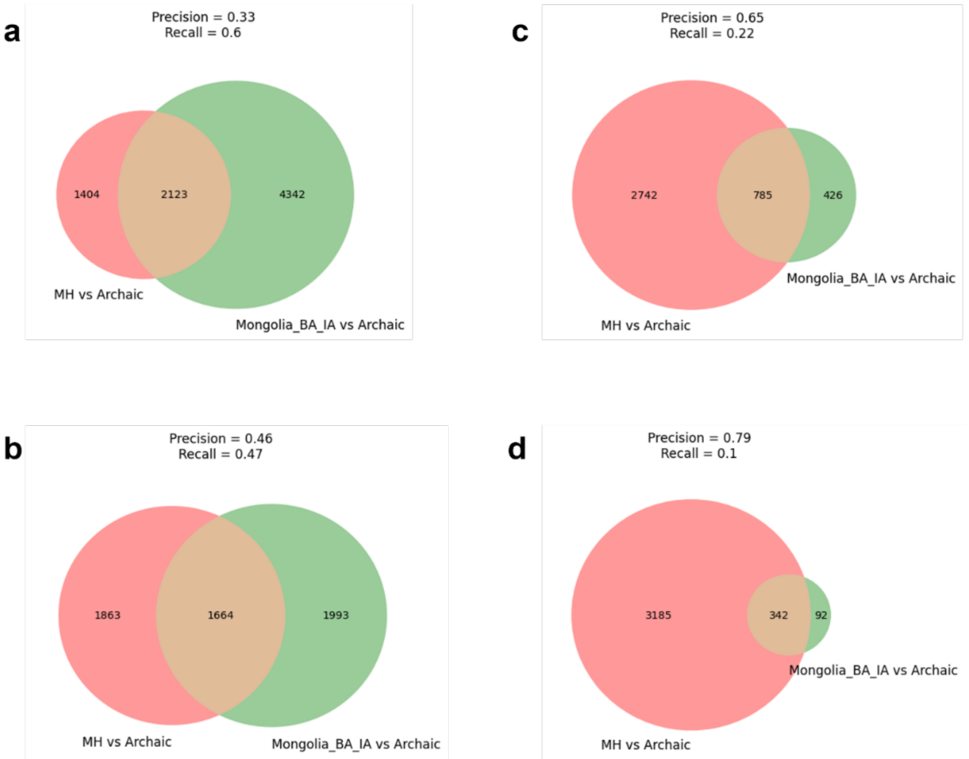

Figure T3.2 *Intersection between DMRs separating modern and archaic humans detected using shotgun samples (red + orange) and using Mongolia_BA_IA (green + orange).* ***a)*** *No filtering;* ***b)*** *filtering using logistic regression with a threshold of 0.2;* ***b)*** *filtering using logistic regression with a threshold of 0.5;* ***c)*** *filtering using logistic regression with a threshold of 0.8.*

Supplementary Text S4

To get a final list of DMRs following the filtration process in comparison of selected populations, we used logistic regression trained on 30% downsampling of Caribbean_CER, $\Delta = 0.4$, and a threshold of 0.5. As expected, filtration considerably reduced the number of DMRs (Supplementary Table S9). For example, the originally 638 DMRs between Mongolia_BA_IA and Caribbean_CER were reduced to only seven following filtration. We tested the number of DMRs within European populations by comparing Eu_BellBeakers to British_N and British_BA combined. We detected 3,196 DMRs, of which 60 remained after filtration. Given the geographic and genetic proximity of these populations, we do not expect to find so many DMRs, providing more evidence to the risk of elevated false detections when using low-quality pooled populations.

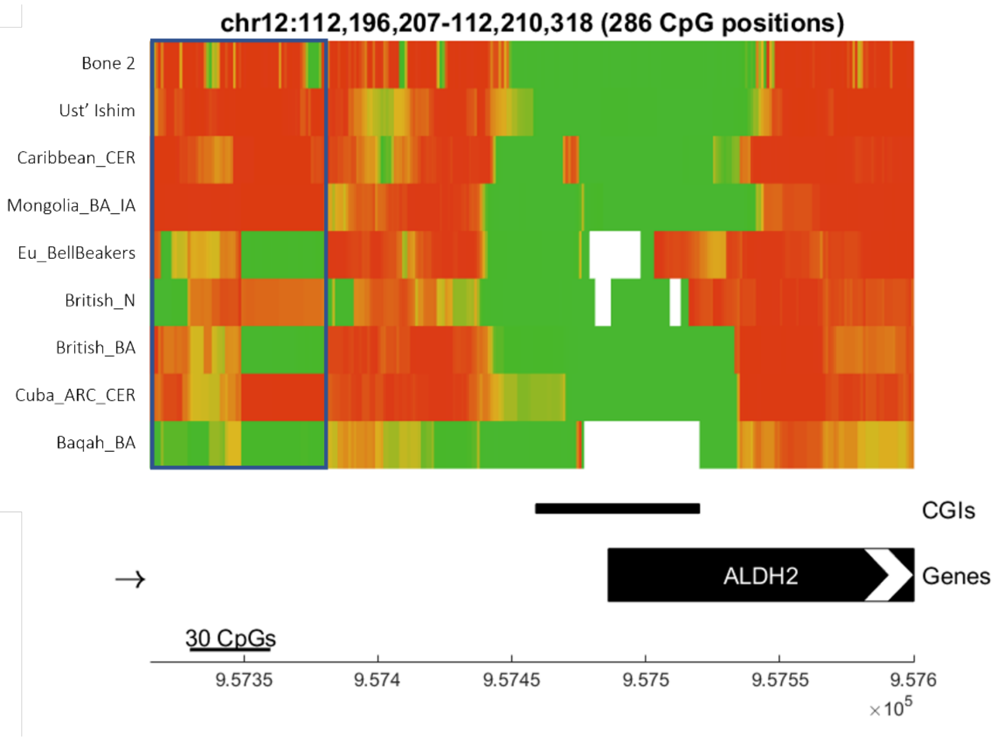

Figure 4.1 *Methylation maps measured (Bone 2) and reconstructed (Ust'-Ishim and the cohorts: Caribbean_CER, Mongolia_BA_IA, Eu_BellBeakers, British_N, British_BA, Cuba_ARC_CER, Baqah_BA) in the promoter of the gene ALDH2. Detected DMR borders are marked in the blue box.*

Per each DMR we calculated its genomic annotation (Supplementary Table S10). Notably, among the 16 DMRs separating Mongolia_BA_IA and European populations, one is in the promoter of the gene ALDH2. This DMR, spanning 65 CpGs and 4,112 base-pairs, shows a very large average methylation difference of 0.57 between the two populations (Figure T2.3). In general, the European populations tend to show hypomethylation in this region, whereas the population of Asian origin, Mongolia_BA_IA, Caribbean_CER and Cuba_ARC_CER, show hypermethylation. Aldehyde dehydrogenase has an important role in alcohol metabolism, and a remarkably higher frequency of acute alcohol intoxication among East Asians than among Caucasians could be related to reduced activity in one of its variants[2,3]. Although the DMR is not in the CpG island that overlaps ALDH2, it is in proximity to the SNP rs2013002 which resides in chromosome 12 in position 112,200,150, within the detected DMR boundaries. The SNP was identified as under positive selection in European populations and is associated with alcohol consumption and blood pressure[4]. In modern east Asian populations, only the variant C is prevalent, whereas in European populations C and T are both prevalent[5].

References

1. Gokhman, D. *et al.* Differential DNA methylation of vocal and facial anatomy genes in modern humans. *Nat Commun* **11**, 1189 (2020).

2. Macgregor, S. *et al.* Associations of ADH and ALDH2 gene variation with self report alcohol reactions, consumption and dependence: an integrated analysis. *Hum Mol Genet* **18**, 580–593 (2009).

3. Oota, H. *et al.* The evolution and population genetics of the ALDH2 locus: random genetic drift, selection, and low levels of recombination. *Ann Hum Genet* **68**, 93–109 (2004).

4. Schaschl, H., Göllner, T. & Morris, D. L. Positive selection acts on regulatory genetic variants in populations of European ancestry that affect ALDH2 gene expression. *Sci Rep* **12**, 4563 (2022).

5. National Institute of Mental Health. https://www.ncbi.nlm.nih.gov/snp/rs2013002. (2022).
